# 762. Conversations that Matter: Developing an AI-Based Training Algorithm to Improve Provider Competence to Work with SGM Youth

**DOI:** 10.1093/ofid/ofad500.823

**Published:** 2023-11-27

**Authors:** Brady Hanshaw, Laura Rachal, Jessica Gross, Zachary Finacchio, Sarah McElroy, Skyler Noble, Joshua Espada, Thomas Shen, A Lina Rosengren

**Affiliations:** Harvard Medical School, Boston, Massachusetts; University of North Carolina at Chapel Hill, Chapel Hill, North Carolina; University of North Carolina at Chapel Hill, Chapel Hill, North Carolina; University of North Carolina at Chapel Hill, Chapel Hill, North Carolina; University of North Carolina at Chapel Hill, Chapel Hill, North Carolina; University of North Carolina at Chapel Hill, Chapel Hill, North Carolina; University of North Carolina at Chapel Hill, Chapel Hill, North Carolina; University of North Carolina at Chapel Hill, Chapel Hill, North Carolina; University of North Carolina at Chapel Hill, Chapel Hill, North Carolina

## Abstract

**Background:**

Cultural incompetence hinders the engagement of sexual and gender minority (SGM) youth in the HIV prevention and care continuum. Improving providers’ cultural competence is critical to reducing HIV health inequities. Medical education lacks opportunity for practice and SGM perspectives. We are developing an AI training app that uses virtual patients and a framework of educational and behavior change theory to increase provider clinical and communication competence with SGM youth. SGM youth and healthcare providers have unique knowledge about provider bias and stigma barriers to care, which can inform intervention content.

**Methods:**

From Twitter's API, 42,213 Tweets were iteratively sampled by 11 SGM youth (the “YAB”) and analyzed thematically. These themes were combined with current clinical care guidelines for SGM youth to generate 111 stigma barriers to care agreed upon by the YAB by consensus. These themes were edited and ranked by importance by the YAB using a modified Delphi method. Using this content as a starting point, we held workshops with providers and the YAB to develop a comprehensive list of scenarios where providers noted deficient training in providing culturally competent care to SGM youth. Prioritized training scenarios selected from this list were developed into detailed scripts for AI virtual patient encounters.

**Results:**

Using a model of social media mining informed by SGM youth and provider collaborative work, training scenarios were developed for integration into a GPT/AI provider training app. Scenarios address topics selected by SGM youth that are particularly salient to this population based on social media conversations around provider stigma and the YAB members’ lived experiences. Scenarios will progress from basic to advanced topics to train providers in culturally appropriate language and foster competent care for SGM youth.

**Conclusion:**

SGM youth have deep insight into their care needs and lived experiences of healthcare trauma. Understanding these care needs and building provider competence is critical to reduce HIV inequities among SGM youth. Our data provide new insight into how interactions between providers and SGM youth may improve. Future research will test the app’s effect on the clinical care behaviors of providers in a randomized control trial.

**Disclosures:**

**All Authors**: No reported disclosures

